# Metformin alleviates the calcification of aortic valve interstitial cells through activating the PI3K/AKT pathway in an AMPK dependent way

**DOI:** 10.1186/s10020-021-00416-x

**Published:** 2021-12-11

**Authors:** Qiao En, Huang Zeping, Wang Yuetang, Wang Xu, Wang Wei

**Affiliations:** Department of Structural Heart Disease, Fuwai Hospital, National Center for Cardiovascular Disease, Chinese Academy of Medical Sciences, Peking Union Medical College, 167 Beilishi Road, Xicheng District, Beijing, 100037 China

**Keywords:** Metformin, Calcific aortic valve disease, Calcification, Aortic valve interstitial cells, PI3K/AKT signaling pathway, Apoptosis

## Abstract

**Background:**

Calcific aortic valve disease (CAVD) is the most prevalent valvular disease worldwide. However, no effective treatment could delay or prevent the progression of the disease due to the poor understanding of its pathological mechanism. Many studies showed that metformin exerted beneficial effects on multiple cardiovascular diseases by mediating multiple proteins such as AMPK, NF-κB, and AKT. This study aims to verify whether metformin can inhibit aortic calcification through the PI3K/AKT signaling pathway.

**Methods:**

We first analyzed four microarray datasets to screen differentially expressed genes (DEGs) and signaling pathways related to CAVD. Then aortic valve samples were used to verify selected genes and pathways through immunohistochemistry (IHC) and western blot (WB) assays. Aortic valve interstitial cells (AVICs) were isolated from non-calcific aortic valves and then cultured with phosphate medium (PM) with or without metformin to verify whether metformin can inhibit the osteogenic differentiation and calcification of AVICs. Finally, we used inhibitors and siRNA targeting AMPK, NF-κB, and AKT to study the mechanism of metformin.

**Results:**

We screened 227 DEGs; NF-κB and PI3K/AKT signaling pathways were implicated in the pathological mechanism of CAVD. IHC and WB experiments showed decreased AMPK and AKT and increased Bax in calcific aortic valves. PM treatment significantly reduced AMPK and PI3K/AKT signaling pathways, promoted Bax/Bcl2 ratio, and induced AVICs calcification. Metformin treatment ameliorated AVICs calcification and apoptosis by activating the PI3K/AKT signaling pathway. AMPK activation and NF-κB inhibition could inhibit AVICs calcification induced by PM treatment; however, AMPK and AKT inhibition reversed the protective effect of metformin.

**Conclusions:**

This study, for the first time, demonstrates that metformin can inhibit AVICs in vitro calcification by activating the PI3K/AKT signaling pathway; this suggests that metformin may provide a potential target for the treatment of CAVD. And the PI3K/AKT signaling pathway emerges as an important regulatory axis in the pathological mechanism of CAVD.

**Supplementary Information:**

The online version contains supplementary material available at 10.1186/s10020-021-00416-x.

## Background

Calcific aortic valve disease (CAVD), the most common valve disease, is characterized by aortic valve calcification and progressive stenosis, leading to left ventricular outflow tract obstruction, arrhythmia, heart failure, and death. The incidence increases with age, and about 12% of the elderly over 75 years old suffer from aortic stenosis (Donato et al. 2020; Osnabrugge et al. 2013). In the initial stage of the disease, it may have no obvious symptoms. However, when the disease progresses to severe aortic stenosis, it may present with symptoms including the sudden onset of chest tightness, angina pectoris, syncope, and dyspnea (Bonow et al. 2016). Without appropriate treatment, the 5-year mortality rate could reach 90% (Kapadia et al. 2015; Leon et al. 2010). At present, most of the patients are already in the advanced stage, when diagnosed with aortic stenosis. Moreover, surgical or transcatheter aortic valve replacement remains the main treatment, but these will lead to many inevitable complications. Patients with mechanical valves need to take anticoagulants for life; the bioprosthetic valve will inevitably deteriorate and lead to the possibility of reoperation less than 15 years (Head et al. 2017). However, no effective drugs can delay or prevent the disease progression of CAVD due to the unclear understanding of the pathological mechanism. Therefore, it is extremely important to discover potential biomarkers, pathways, and drugs through basic experimental research.

The aortic valve comprises three layers of tissue structure, including the laminae fibrosa, ventricularis, and the laminae spongiosa located between the fibrosa and the ventricularis (Chen and Simmons 2011). The most important cellular population includes aortic valve interstitial cells (AVICs) and valve endothelial cells. In normal valves, AVICs are mainly fibroblast-like phenotype and express vimentin, while in calcific valves, AVICs are mainly phenotypically similar to the osteoblast (Rutkovskiy et al. 2017). Osteogenic differentiation of AVICs plays an important role in the occurrence and development of CAVD (Goody et al. 2020). Many biomarkers and signaling pathways are associated with aortic calcification and osteogenic differentiation of AVICs. The NOTCH 1 gene mutation is related to CAVD initiation and progression (Garg et al. 2005); transforming growth factor-β, bone morphogenetic protein (BMP), Wnt, and AKT, etc*.* are also involved in the pro-osteogenic activation of AVICs (Dutta and Lincoln 2018; Yang et al. 2009). Previous studies showed that AKT was significantly downregulated in calcific valves, and AKT inhibition could promote calcific nodule formation in AVICs, promote the apoptosis of AVICs and secretion of inflammatory factors, including IL6, IL8, IL1β, and MCP-1, etc. (Deng et al. 2016; El Husseini et al. 2014; Zhu et al. 2019). However, the role of the PI3K/AKT signaling pathway in the pathological mechanism of CAVD was poorly understood at present.

Metformin, a derivative of biguanide, has an excellent hypoglycemic effect and remains a first-line treatment for type 2 diabetes mellitus (Lv and Guo 2020; Rena and Lang 2018). Moreover, increasing studies show that metformin has beneficial effects in a variety of diseases, including multiple cancers (Schulten 2018), multiple sclerosis (Dziedzic et al. 2020), obesity, atherosclerosis, vascular calcification (Mary et al. 2017), and cardiac ischemia–reperfusion (Higgins et al. 2019). Metformin can mediate the expression of multiple biomarkers and pathways, such as AMPK, endothelial nitric oxide synthase (eNOS), NF-κB, and the PI3K/AKT signaling pathways, etc*.*, thereby exerting anti-inflammatory, anti-apoptotic, and anti-oxidant stress effects, etc. (Higgins et al. 2019; Lv and Guo 2020). Metformin can inhibit the production of adenosine triphosphate and promote the production of adenosine monophosphate and adenosine diphosphate, thereby activating AMPK, which is the key regulator for metformin to exert beneficial effects in various diseases (Zhou et al. 2001). However, few basic and clinical studies reported the role of metformin in the treatment of CAVD. This study aims to investigate whether metformin can inhibit the calcification of AVICs by activating the PI3K/AKT signaling pathway in an AMPK-dependent manner.

## Materials and methods

### Potential signaling pathways in CAVD by using bioinformatics analysis

We screened 4 transcriptome datasets about CAVD from the Gene Expression Omnibus database (https://www.ncbi.nlm.nih.gov/geo/) (Barrett et al. 2013), including GSE12644 (Bossé et al. 2009), GSE51472 (Ohukainen et al. 2015), GSE77287, and GSE83453 (Guauque-Olarte et al. 2016). After excluding bicuspid aortic valves, 26 normal and 27 calcific aortic valve tissues were included. The screening criteria for differentially expressed genes (DEGs) were: |log2 (fold change) |> 0.585 and adjusted p value < 0.05. Finally, to screen potential signaling pathways, Kyoto Encyclopedia of Genes and Genomes (KEGG) pathway enrichment analysis was performed using the Database for Annotation, Visualization, and Integrated Discovery online gene functional classification tool (https://david.ncifcrf.gov/), with the cut-off criteria of p value < 0.05 (Jiao et al. 2012). And the ggplot2 package of the R software was used to visualize the KEGG analysis result.

### Human tissue samples

Calcific aortic valves were obtained from eight CAVD patients undergoing aortic valve replacement surgery; nine control aortic valve samples were from patients with aortic valve prolapse or severe aortic valve regurgitation, and no aortic valve thickening or calcification was confirmed by transthoracic echocardiography and CT. Exclusion criteria included congenital bicuspid aortic valves, rheumatic heart disease, and infective endocarditis. All valve tissues were obtained in a sterile environment. The aortic valve was divided into 2–3 parts, one part was stored in liquid nitrogen for protein extraction, and the other was stored in 4% paraformaldehyde (PFA) for histopathological analysis, and one part was stored in M199 medium for AVICs isolation.

### Histological and immunohistochemical (IHC) staining

The tissue was fixed with 4% PFA for 24 h and embedded in paraffin wax, and then cut into 4 μm sections. Hematoxylin–eosin (HE), Masson’s trichrome, and Von Kossa staining were performed in accordance with the manufacturer’s instructions. For IHC staining, sodium citrate solution (pH 6.0) was used to perform antigen retrieval; after washing with phosphate buffer solution (PBS), the peroxidase blocking agent and bovine serum albumin (BSA) were added to block the endogenous peroxidase activity and antigen. The tissue sections were incubated with the following primary antibodies, including p-AMPKα (2535, Cell Signaling Technology, 1:100), p-AKT (4060, Cell Signaling Technology, 1:100), and Bax (5023, Cell Signaling Technology, 1:100) at 4 °C overnight. After rewarming and rinsing with PBS, the sections were incubated with the corresponding secondary antibodies for 1 h at 37 °C. Moreover, DAB chromogenic solution (ab64238, Abcam, UK) was used for color development. All histopathological sections were imaged using the 3DHISTECH panoramic SCAN instrument.

### Terminal deoxynucleotidyl transferase-mediated dUTP nick-end labeling (TUNEL) assay

Apoptosis assays were performed using a colorimetric TUNEL apoptosis assay detection kit (C1098, Beyotime, China) according to the manufacturer’s instructions. Briefly, after the tissue section were rehydrated and rinsed with distilled water, the proteinase K solution was used for antigen retrieval. Then the peroxidase blocking reagent was used to block the endogenous peroxidase. The sections were incubated in the TUNEL reaction mixture at 37 °C for 60 min in the dark, then incubated with Streptavidin-HRP for 30 min at room temperature. Chromogenic detection was performed with DAB chromogenic reagent, and finally, the tissue sections were stained with hematoxylin staining solution. 3DHISTECH panoramic SCAN instrument was used for image acquisition; TUNEL-positive cell nuclei were stained brown, and the negative normal cell nuclei were stained blue. Apoptosis index was calculated as the percentage of TUNEL-positive cells to the total number of cells.

### Cell isolation, culture, and treatment

Human AVICs were isolated from normal non-calcific aortic valves by using the collagenase I digestion method. First, aortic valves were digested using 2 mg/ml collagenase I (Sigma-Aldrich) for 10 min at 37 °C and swabbed with a sterile swab to wipe off the endothelial cells. After washing three times with PBS, the valves were minced and digested with 2 mg/ml collagenase I at 37 °C for 3 h. The suspension was spun at 1000 rpm for 10 min to precipitate cells. Cells were resuspended using M199 medium (M4530, 1 g/l glucose, Sigma-Aldrich) supplemented with 10% fetal bovine serum (FBS), 100 U/ml penicillin, and 100 μg/ml streptomycin and plated onto a 25 cm^2^ sterile flask in a humidified atmosphere with 5% CO2 at 37 °C. We only use cells at 3 to 7 passages for experiments. To induce AVICs to differentiate into osteoblast phenotype, after the AVICs reaching 70–80% confluence, we used the phosphate medium (PM) containing M199 with 10% FBS, 2 mM NaH_2_PO_4_ (Sigma-Aldrich), and 50 μg/ml l-ascorbic acid (Sigma-Aldrich) for indicated days. To verify the effect of metformin on AVICs calcification, we used three different concentrations of metformin (10 μM, 100 μM, and 1 mM), AMPK activator (AICAR, 500 μM, Selleck, USA), AMPK inhibitor (compound C, CC, 10 μM, Selleck, USA), NF-κB inhibitor (Pyrrolidinedithiocarbamate ammonium, PDTC, 10 μM, MedChemExpress, USA), and AKT inhibitor (MK2206, 5 μM, Selleck, USA) to treat AVICs. The culture medium was refreshed every three days.

### Immunofluorescence (IF) staining

To determine the phenotype of AVICs and verify the effect of metformin on the expression levels of p-AKT and osteopontin (OPN), we performed IF staining experiments. AVICs were seeded in 4-well chamber slides and cultured with normal medium, PM with or without metformin, AICAR, CC, and MK2206 for 72 h. After being fixed with 4% PFA for 15 min, AVICs were permeabilized with 0.1% Triton in PBS and blocked with 5% BSA. AVICs were incubated with the following primary antibodies: α-SMA (ab5694, Abcam, UK, 1:300), vimentin (ab8978, Abcam, UK, 1:500), p-AKT (4060, Cell Signaling Technology, 1:100), and osteopontin (ab8448, Abcam, UK, 1:500) at 4 °C overnight. After washing with PBS, AVICs were incubated with the corresponding fluorescent secondary antibodies at 37 °C for 1 h; 4′,6-diamidino-2-phenylindole (DAPI) was used to counterstain the nucleus. The IF staining images were obtained using a laser scanning confocal microscope (SP8, Leica, Germany).

### Cell viability assay

Cell viability was detected using the Cell Counting Kit-8 (CCK8) assay (C0039, Beyotime, China). AVICs were seeded into a 96-well plate (4000 cells/well) with 100 μl medium. After cells adhered to the wall, AVICs were cultured with the corresponding reagents for 72 h; 10 μl of CCK8 reagent was added to each well and continued to incubate for 4 h. Medium without cells was used as a negative control. At least six replicates were set for each group. The optical density (OD) value of each well was detected using a microplate reader at the wavelength of 450 nm.

### Measurement of cellular reactive oxygen species (ROS)

The level of cellular ROS was measured using a ROS detection kit (S0033, Beyotime, China). Briefly, AVICs were seeded in a 96-well plate (4000 cells/well), cultured with PM and three concentrations of metformin for 72 h. Moreover, 100 μl of 10 μM DCFH-DA reagent was added to each well and incubated at 37 ℃ for 30 min. Cells cultured in normal medium were used as a negative control. Excessive dyes were removed using M199 medium without FBS. A fluorescence microplate reader was used to measure the fluorescence intensity value, with excitation wavelength of 488 nm and emission wavelength of 525 nm.

### Enzyme-linked immunosorbent assay (ELISA)

The levels of IL6 (KE00007, Proteintech, China), IL8 (KE00006, Proteintech, China), and MCP-1 (KE00091, Proteintech, China) in the supernatant of AVICs were determined by ELISA. To determine the effect of metformin on PM-induced inflammatory response, we collected the supernatant of AVICs cultured in normal medium, PM, and three concentrations of metformin for 72 h. The ELISA experiment was carried out according to the manufacturer’s instructions, and the OD value was measured using a microplate reader at 450 nm.

### AKT gene silencing

To knock down AKT, cells were seeded in 6-well plates. When the confluence reached 60–70%, AVICs were transfected with the siRNA against AKT1 in Opti-MEM medium (Gibco) using Lipofectamine RNAiMAX reagent (Invitrogen, USA) according to the manufacturer’s instructions. Three distinct AKT1 siRNAs were transfected to knock down the expression of AKT, and the sequences were shown in Additional file 1: Table S2. Quantitative real time-PCR (qRT-PCR) and western blot (WB) assays were carried out to determine the knockdown efficiency. AVICs were transfected with the siRNA for 12 h, and the medium was changed to PM with or without metformin. For long-term AVICs calcification inducing, cells were re-transfected at day three.

### RNA preparation and qRT-PCR

Total RNA was extracted from AVICs using Trizol reagent (1596026, Invitrogen, USA) according to the manufacturer’s instructions. RNA was reverse transcribed into cDNA using PrimeScript™ RT reagent Kit with gDNA Eraser (RR047A, Takara, Japan). And qRT-PCR amplification was performed using the Hiseff® qPCR SYBR Green Master Mix (11202ES08, Yeason, China) on an ABI QuantStudio 3 (ThermoFisher, USA) instrument. The amplification conditions were set as denaturation at 95 °C for 5 min and 40 amplification cycles (15 s at 95 °C and 1 min at 60 °C). The primer sequences are as follows: AKT1 (forward 5′-AGCGACGTGGCTATTGTGAAG-3′ and reverse 5′-GCCATCATTCTTGAGGAGGAAGT-3′), GAPDH (forward 5′-ACAACTTTGGTATCGTGGAAGG-3′ and reverse 5′-GCCATCACGCCACAGTTTC-3′), and GAPDH was used as an internal control. The 2^−ΔΔCt^ method was used to calculate the fold change of AKT1 mRNA.

### Western blot assays

After 72 h of culturing with different treatments, AVICs were lysed using a commercial sample buffer containing a protease and phosphatase inhibitor cocktail (Roche, Switzerland). The protein concentration was determined using a BCA Protein Assay Kit (P0012, Beyotime, China); the total protein was separated using NuPAGE 4–12% Bis–Tris Gel (NP0321BOX, Invitrogen, USA) and then transferred to 0.2 μm polyvinylidene fluoride (PVDF) membranes. Non-specific binding was blocked with 5% non-fat milk in TBST solution (50 mM Tris/HCL, pH 7.6, 150 mM NaCl and 0.1% (vol/vol) Tween-20) at room temperature for 1 h. The membrane was then incubated with corresponding primary antibodies overnight at 4 °C. After washing with TBST, the membranes were incubated with appropriate secondary antibodies for 1 h at room temperature. Antigen–antibody specific binding bands were visualized using the chemiluminescence Lumi-Light Western Blotting Substrate (WBKLS0500, Millipore). Band density was analyzed using the ImageJ software (National Institutes of Health, USA). GAPDH was used as an internal control to normalize protein bands. The primary antibodies used in this study were as follows: GAPDH (AF1186, Beyotime, China, 1:3000), AMPKα (5831, Cell Signaling Technology, 1:1000), p-AMPKα (Thr172) (2535, Cell Signaling Technology, 1:1000), PI3K p110α (4249, Cell Signaling Technology, 1:1000), AKT (4685, Cell Signaling Technology, 1:1000), p-AKT (Ser473) (4060, Cell Signaling Technology, 1:1000), eNOS (32,027, Cell Signaling Technology, 1:1000), p-eNOS (Ser1177) (ab184154, Abcam, 1:500), IKKβ (8943, Cell Signaling Technology, 1:1000), p-IKKα/β (Ser176/180) (2697, Cell Signaling Technology, 1:1000), NF-κB (8242, Cell Signaling Technology, 1:1000), p-NF-κB (Ser536) (3033, Cell Signaling Technology, 1:1000), BMP2 (66383–1-Ig, Proteintech, 1:500), osteopontin (ab8448, Abcam, 1:1000), Bax (5023, Cell Signaling Technology, 1:1000), Bcl2 (4223, Cell Signaling Technology, 1:500), caspase3 (9662, Cell Signaling Technology, 1:1000), and cleaved caspase 3 (9661, Cell Signaling Technology, 1:500).

### Alizarin red S (ARS) staining

To visualize calcium deposition of AVICs, ARS staining assays were performed. The cells were seeded in 12-well plates. After reaching 70–80% confluence, cells were incubated with different interventions for 7 days. AVICs were washed with PBS solution three times, fixed with 4% PFA for 15 min, and then incubated with 2% ARS dye solution (pH 4.0–4.2) (ab146374, Abcam, UK) for 15 min. Moreover, excessive ARS dye was removed with distilled water, and the stained cells were photographed. The red staining represents calcium nodule formation. To quantify calcium deposits, we incubated the stained cells with a 10% aqueous solution of cetyl-pyridinium chloride (Sigma-Aldrich) to release the ARS dye from the extracellular matrix, and the absorbance was measured using a spectrophotometer at 560 nm.

### Statistical analysis

All values were presented as mean ± standard deviation (SD), and statistical analysis was performed using SPSS software 25.0 (IBM, Chicago, USA). After determining the normal distribution using the Shapiro–Wilk test, data analysis was performed using Student’s t-test or non-parametric test such as Mann–Whitney. For the comparative analysis of multiple groups, we used one-way ANOVA with Bonferroni post hoc test. Statistical analysis with significant difference was set at p < 0.05.

## Results

### DEGs and KEGG pathway enrichment analysis by bioinformatics analysis

Four mRNA microarray datasets, including GSE12644, GSE51472, GSE77287, and GSE83453, were used for bioinformatics analysis; and a total of 26 normal and 27 calcific aortic valves were included in these datasets. We screened 227 DEGs, including 142 up-regulated and 85 down-regulated genes. The heatmap (Additional file 1: Fig. S1) indicated the expression of each gene. KEGG pathway analysis reflected that PI3K/AKT and NF-κB signaling pathways might be closely associated with the occurrence and development of CAVD (Additional file 1: Fig. S2). The results of more bioinformatics analyses were shown in the Additional file 1: Fig. S3, S4.

### Valve histopathology and protein expression change in calcific valves

**Fig. 1:**
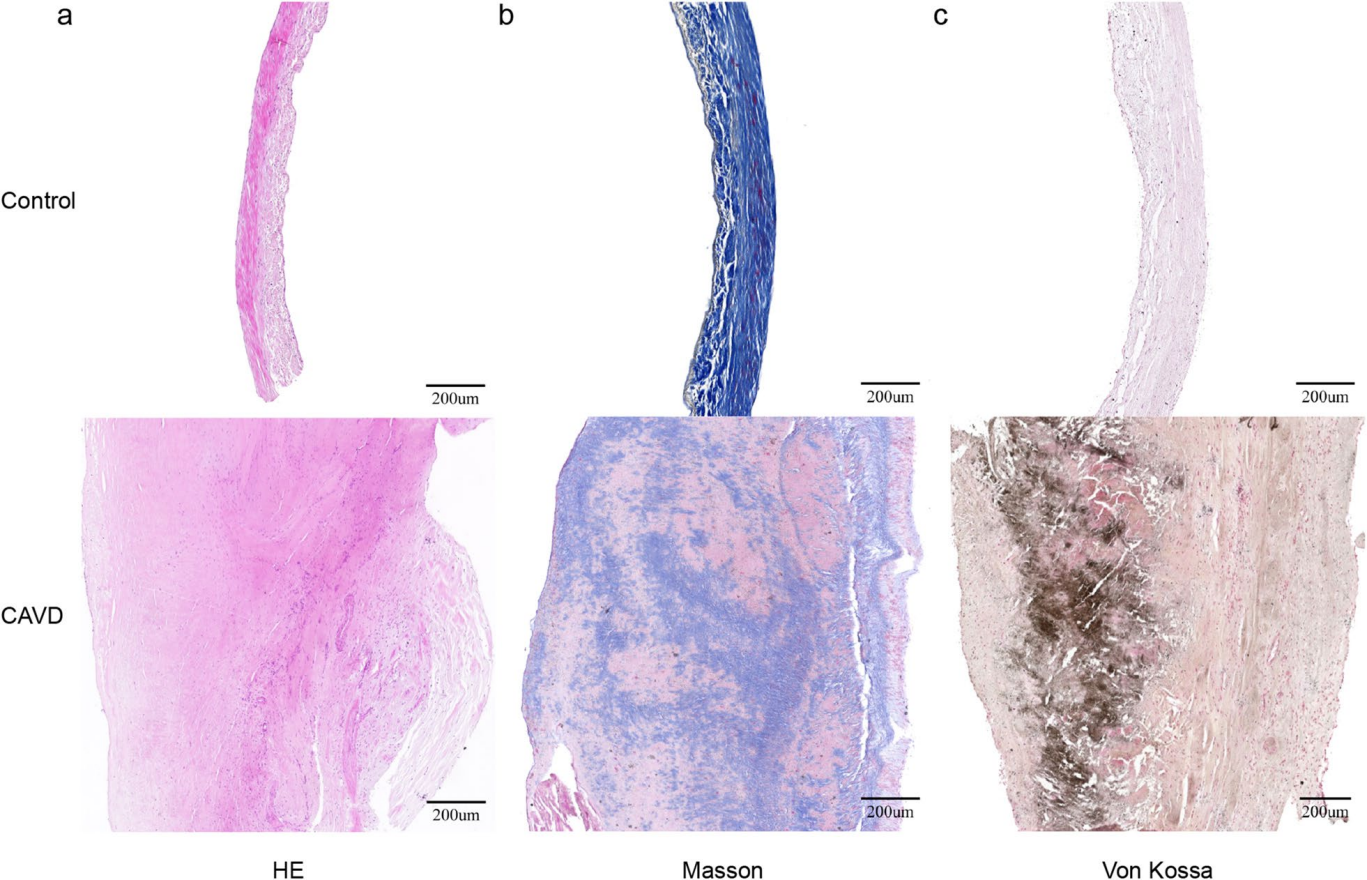
Histochemical staining of aortic valves. **a** and **b** Hematoxylin–eosin (HE) and Masson’s trichrome staining showing valve thickening and collagen fiber hyperplasia; **c** Von Kossa staining showing calcium deposition in valves, black indicates calcium deposition. Scale bar, 200 μm; *CAVD* calcific aortic valve disease

**Fig. 2:**
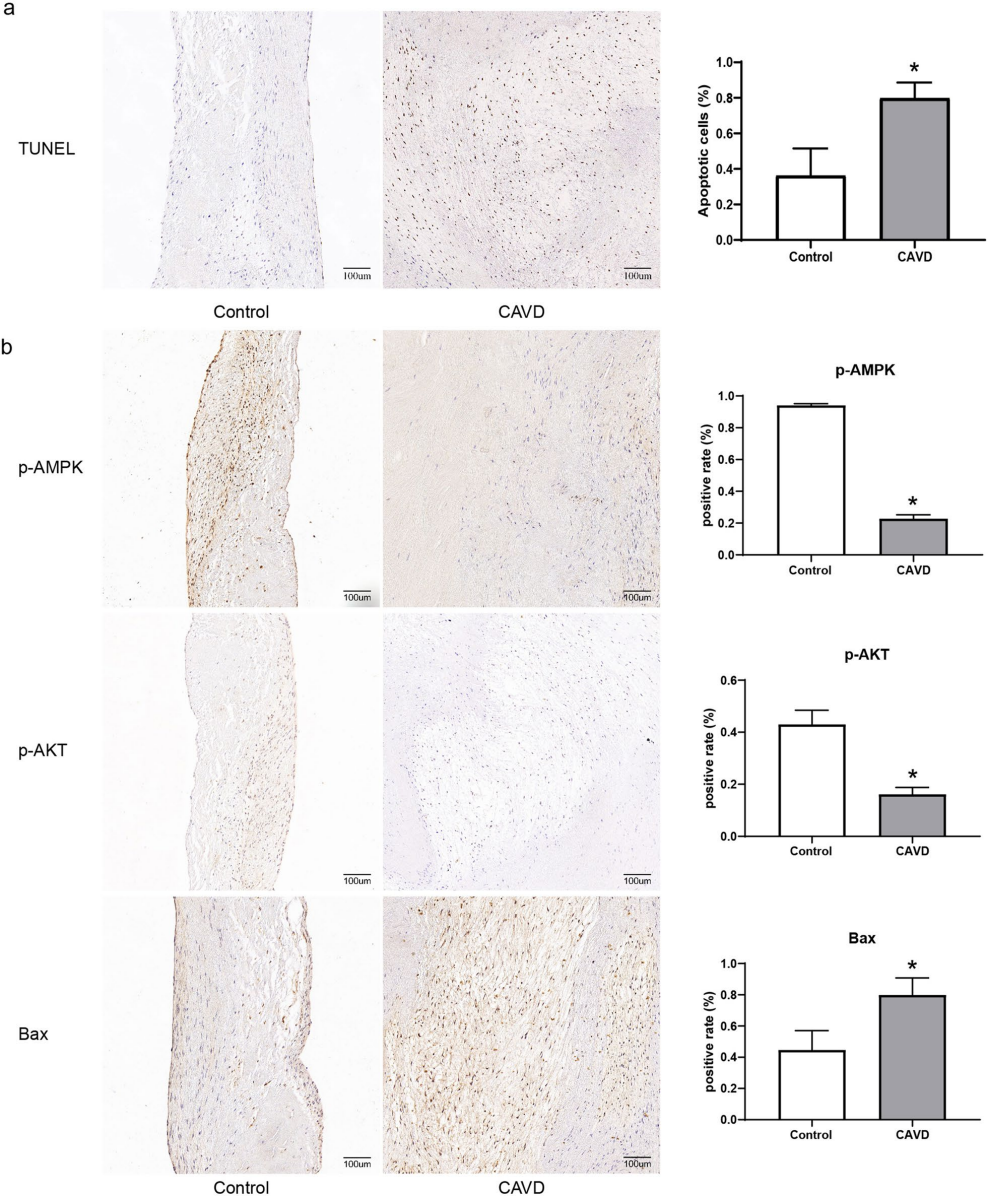
TUNEL and immunohistochemical staining of aortic valves. **a** Cell apoptosis was detected by TUNEL staining; **b** immunohistochemical evaluation of p-AMPK, p-AKT, and Bax expression in control (n = 9) and calcific (n = 8) aortic valves. Values represent the percentage of positive cells in the total number of cells in the sample; Scale bar, 100 μm; CAVD, calcific aortic valve disease; *p < 0.05 versus control group (Student’s t-test)

**Fig. 3:**
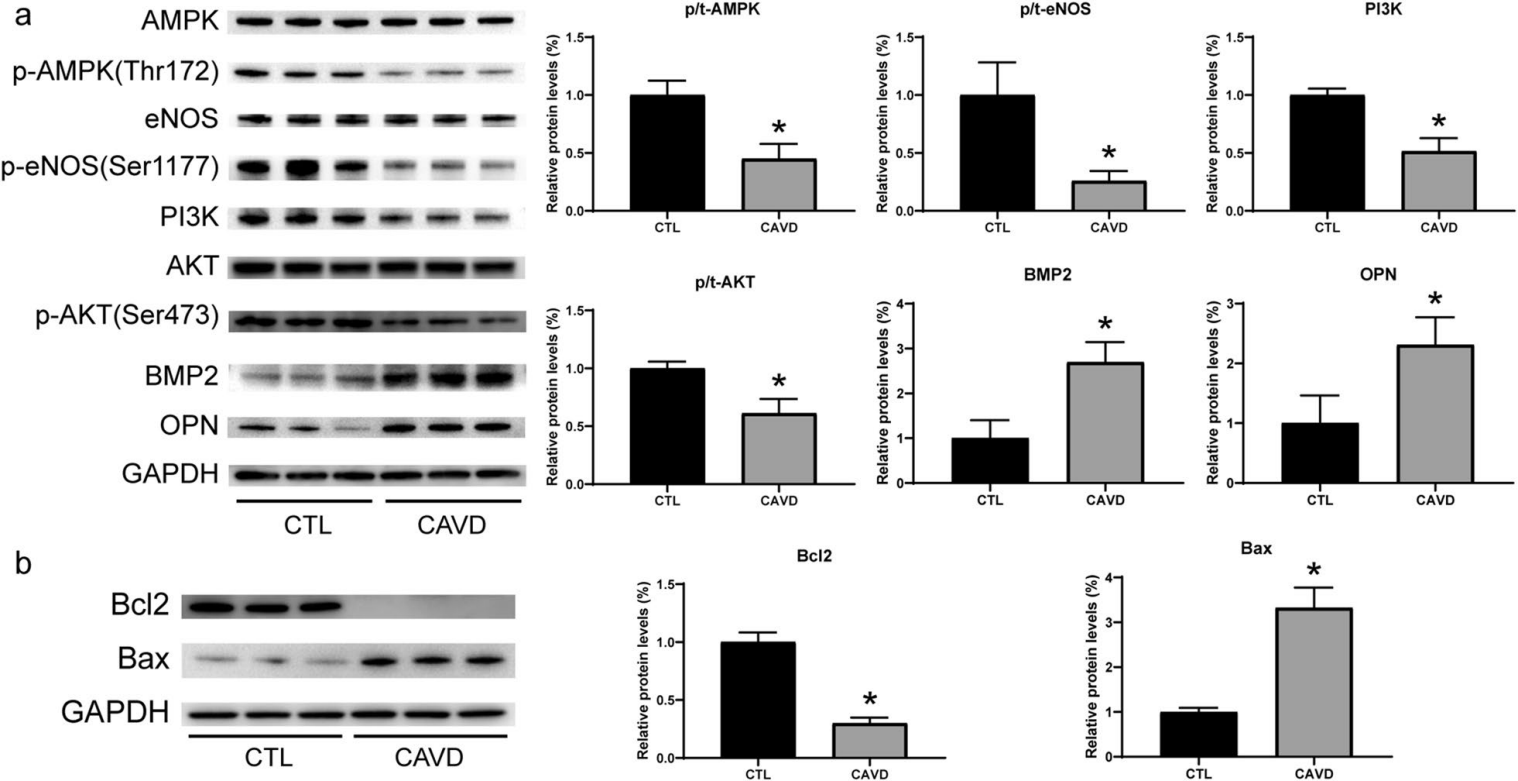
Protein expression level change between control and calcific aortic valves. **a** Protein expression levels of p-AMPK (Thr172), p-AKT (Ser473), PI3K, p-eNOS (Ser1177), BMP2, and OPN of control (n = 9) and calcific (n = 8) valves were determined by WB and quantification analysis; **b** protein expression levels of Bax and Bcl2 were determined by WB and quantification analysis. *CTL* control; *CAVD* calcific aortic valve disease; *p < 0.05 versus control group (Student’s t-test)

Nine normal and eight calcific aortic valve specimens were included in this study (Additional file 1: Table S1). The histological staining results of these valves are shown in Fig. 1. Compared with normal valves, calcific valves showed extensively hyperplastic collagen fibers and neovascularization in HE (Fig. 1a) and Masson staining (Fig. 1b), increased calcific deposition in Von Kossa staining (Fig. 1c), and obvious apoptosis in TUNEL staining (Fig. 2a). IHC staining showed that the expression of phosphorylated AMPK and AKT was significantly down-regulated, and the expression of Bax was significantly up-regulated in calcific valves (Fig. 2b). WB assays showed that the expression of PI3K, AKT, eNOS, AMPK, and Bcl2 were all down-regulated, but BMP2, OPN, and Bax were significantly up-regulated in calcific valves (Fig. 3).

### Isolation and validation of AVICs

To investigate the effect of metformin on AVICs calcification, we isolated AVICs from non-calcific aortic valves. We next performed IF staining to determine the isolated AVICs, and AVICs showed positive Vimentin and weakly positive α-SMA staining (Additional file 1: Fig. S5).

### Metformin inhibits ROS production and inflammatory factor secretion of AVICs induced by PM

**Fig. 4:**
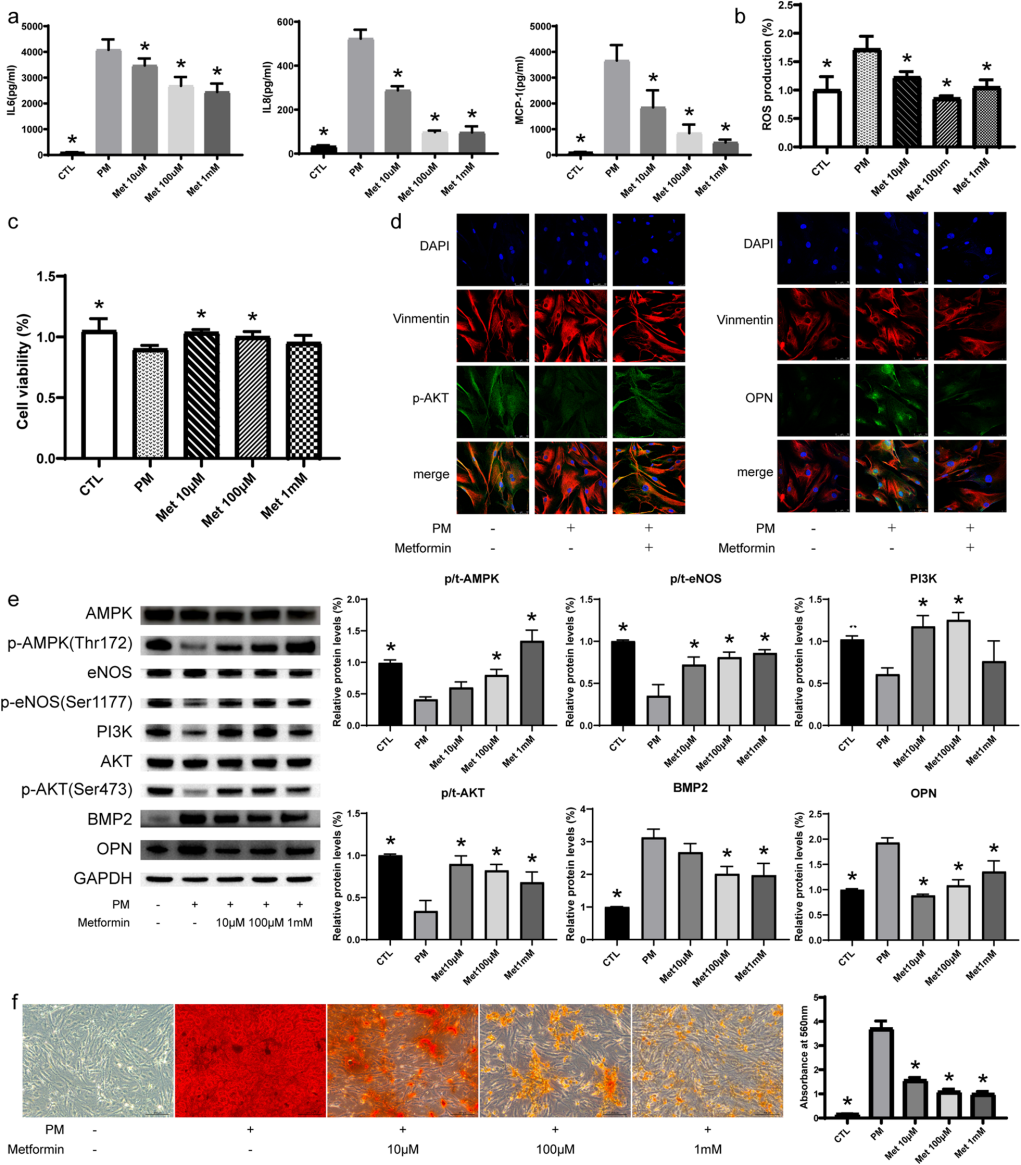
Metformin alleviates aortic calcification by activating the PI3K/AKT signaling pathway in a concentration-dependent manner in vitro. **a** Metformin inhibits inflammatory factor secretion including IL6, IL8, and MCP-1 in cell supernatant, after treatment with phosphate medium (PM) with or without metformin for 72 h as determined by ELISA; **b** ROS production was detected and quantified after PM treatment with or without metformin for 72 h; **c** Cell viability was detected by CCK8 after treatment with PM with or without metformin for 72 h; **d** Immunofluorescence staining images of p-AKT and OPN expression in AVICs after PM treatment with or without 100 μM metformin for 72 h, Scale bar, 50 μm; **e** The protein expression of p-AMPK (Thr172), p-AKT (Ser473), PI3K, p-eNOS (Ser1177), BMP2, and OPN in AVICs after PM treatment with or without metformin for 72 h as determined by WB; **f** Calcium deposition were detected by ARS Staining after various treatments for 7 days, Scale bar, 200 μm. n = 6 per group. CTL, control; Met, metformin; *p < 0.05 versus PM group (one-way ANOVA with Bonferroni post hoc test)

Inflammation is closely associated with the occurrence and development of CAVD. Previous studies showed that inflammatory cells existed in the aortic valve before the formation of calcific nodules; PM can induce AVICs to secrete inflammatory factors, including IL6, IL8, and MCP-1. To evaluate whether metformin can inhibit PM-induced inflammatory response, we used PM containing three different concentrations of metformin (10 μM, 100 μM, and 1 mM). ELISA experiments showed that metformin inhibited the secretion of inflammatory factors in a dose-dependent way (Fig. 4a). Moreover, ROS might also be involved in the pathological mechanism of CAVD. PM could induce the production of ROS in AVICs; ROS detection experiments showed that metformin could attenuate the production of ROS, and 100 μM metformin had the greatest inhibitory effect (Fig. 4b).

### Metformin attenuates apoptosis and promotes proliferation of AVICs

Apoptosis plays an important role in the occurrence and development of CAVD. After 72 h of induction, PM could induce apoptosis and inhibit the proliferation of AVICs. In order to explore the role of metformin in inhibiting apoptosis, we used three concentrations of metformin for treatment. The results showed that metformin could inhibit the protein levels of apoptotic markers, Bax and Cleaved caspase 3, and increase the expression of pro-survival protein Bcl2, as confirmed by WB assays. Moreover, metformin reached the maximum inhibitory effect at the concentration of 100 μM (Additional file 1: Fig. S6). Moreover, CCK8 experiments showed that metformin could promote the proliferation of AVICs (Fig. 4c).

### Metformin inhibits the osteogenic differentiation of AVICs through the PI3K/AKT signaling

PM could induce AVICs to differentiate from fibroblast-like to osteoblast-like phenotype, increase the protein expression of osteogenic markers, such as BMP2 and OPN, down-regulate the expression of the PI3K/AKT signaling pathway, and reduce the protein expression of phosphorylated AMPK and eNOS. To further confirm whether metformin can inhibit PM-induced osteogenic differentiation of AVICs through the PI3K/AKT signaling pathway, we used three concentrations of metformin for treatment; as expected, metformin treatment significantly inhibited the protein level of BMP2 and OPN, which coincided with an increase in the protein level of PI3K, phosphorylated AKT, eNOS, and AMPK, as confirmed by IF and WB assays (Fig. 4d, e). These results preliminarily indicate that metformin could inhibit the osteogenic differentiation of AVICs by activating the PI3K/AKT signaling pathway and AMPK.

### Metformin attenuates calcification induced by PM

Calcium deposition increased significantly in AVICs after being cultured with PM for seven days. Then we explored the effect of metformin on AVICs in vitro calcification and found that metformin treatment could significantly reduce calcium deposition and calcific nodule formation. And 100 μM metformin could reach a good inhibitory effect on calcium nodule formation, which was not significantly different with the inhibitory effect of 1 mM metformin (Fig. 4f). Taken together, we determined that metformin exerted an anti-calcification effect in human AVICs, and metformin reached significant inhibition at a concentration of 100 μM.

### The anti-calcification and pro-proliferation effects of metformin depend on AMPK activation

**Fig. 5:**
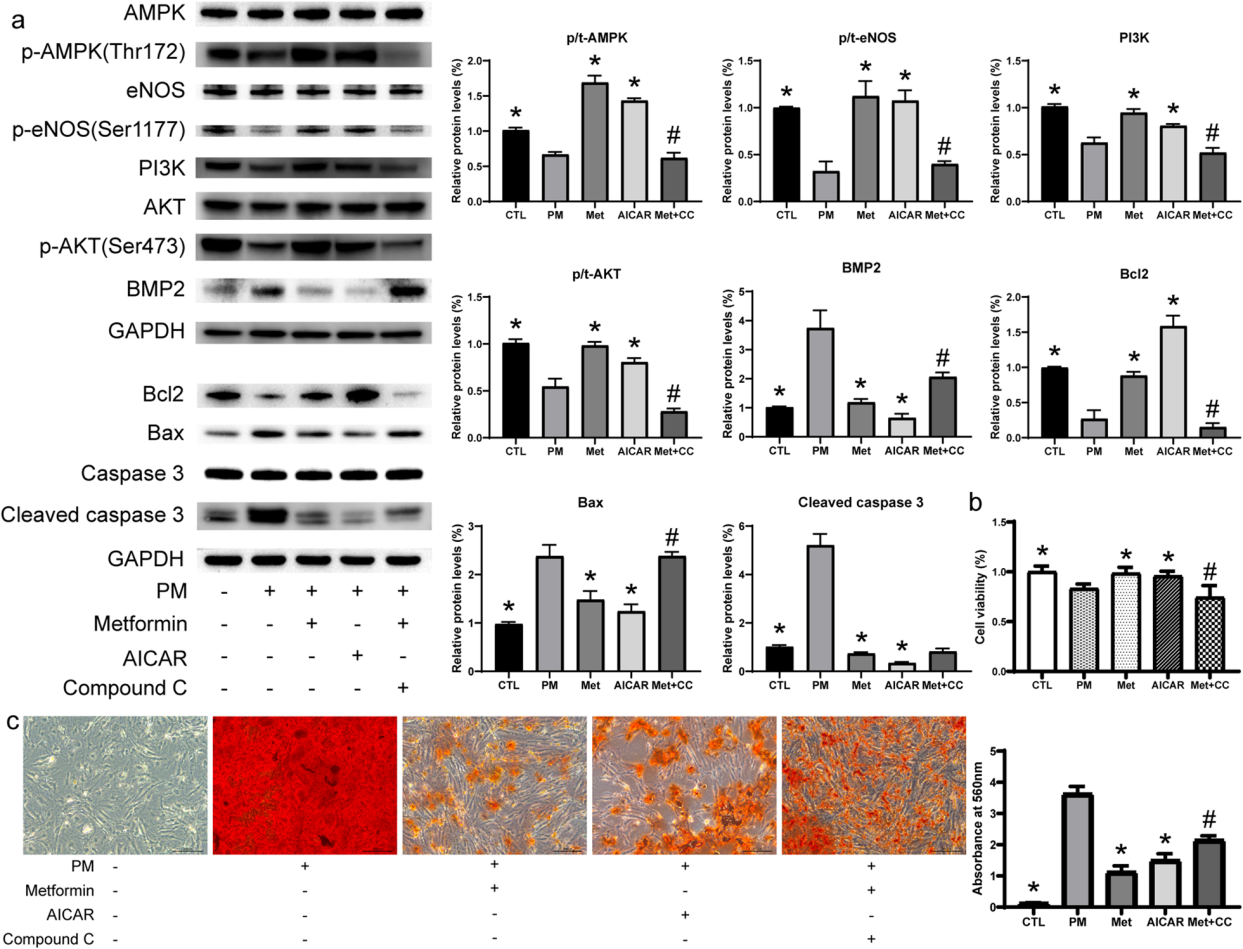
AMPK inhibitor ameliorates the anti-calcification and anti-apoptosis effect of metformin on AVICs. **a** Protein expression levels of the PI3K/AKT and apoptosis pathway in AVICs after various treatments with metformin (100 μM), AMPK activator (AICAR, 500 μM), and AMPK inhibitor (Compound C, CC, 10 μM) for 72 h as determined by WB; **b** cell viability was detected by CCK8 after various treatments for 72 h; **c** calcium deposition were detected by ARS Staining after various treatments for 7 days, Scale bar, 200 μm. n = 6 per group. *CTL* control; *PM* phosphate medium; *Met* metformin; *p < 0.05 versus PM group; ^#^p < 0.05 versus Met group (one-way ANOVA with Bonferroni post hoc test)

To verify whether AMPK is the key regulator for metformin to exert an anti-calcification effect, we used AICAR (500 μM) and CC (10 μM) to culture AVICs. Cell proliferation experiments showed that AICAR could promote cell proliferation, and the proliferation effect of metformin was inhibited by CC (Fig. 5b). Moreover, AICAR could inhibit the expression of apoptotic proteins and up-regulate the expression of Bcl2 protein; however, CC inhibited the anti-apoptotic effect of metformin. We also found that 500 μM AICAR promoted the protein levels of PI3K, phosphorylated AMPK, AKT, and eNOS, and inhibited the levels of osteogenic markers, which is consistent with the effect of metformin; however, 10 μM CC could attenuate the effect of metformin (Fig. 5a). To further verify the effect of AMPK, we performed ARS staining to quantify calcium deposition. The ARS results showed that 500 μM AICAR significantly inhibited PM-induced calcific nodule formation and calcium deposition, and 10 μM CC significantly inhibited the anti-calcification effect of metformin (Fig. 5c). AMPK was a key regulator in the anti-calcification effect of metformin.

### The anti-calcification effect of metformin is related to NF-κB inhibition

**Fig. 6:**
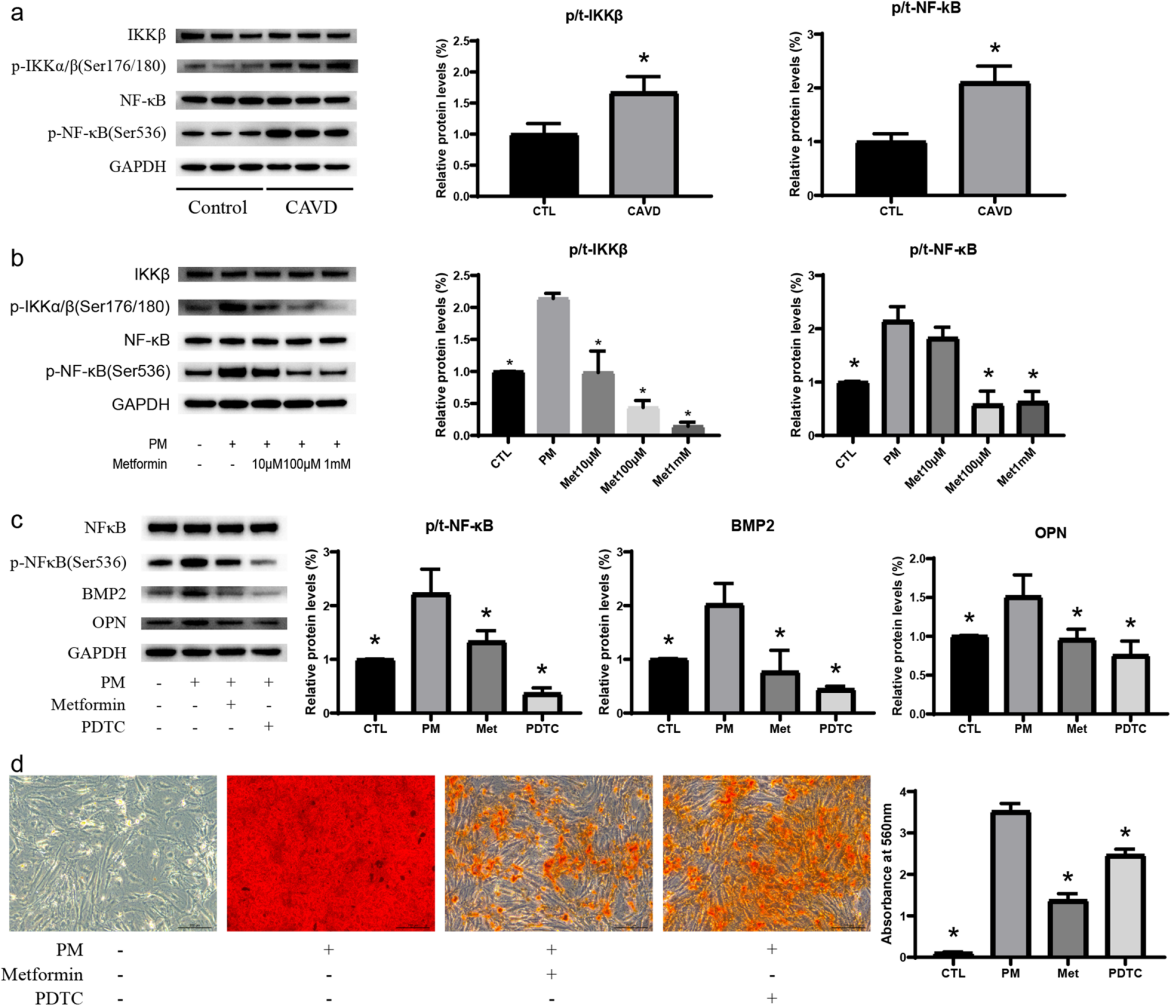
The anti-calcification effect of metformin is related to NF-κB inhibition. **a** Protein expression levels of p-IKKα/β (Ser176/180) and p-NF-κB (Ser536) of control (n = 9) and calcific (n = 8) valves were determined by WB, *p < 0.05 versus control group (Student’s t-test); **b** the protein expression of p-IKKα/β (Ser176/180) and p-NF-κB (Ser536) in AVICs after PM treatment with or without metformin for 72 h as determined by WB, n = 3 per group; **c** protein expression levels of p-NF-κB (Ser536), BMP2, and OPN in AVICs after various treatments with metformin (100 μM) and NF-κB inhibitor (PDTC, 10 μM) for 72 h as determined by WB, n = 3 per group; d, Calcium deposition were detected by ARS Staining after various treatments for 7 days, Scale bar, 200 μm, n = 6 per group. *CTL* control; *PM* phosphate medium; Met, metformin; CAVD, calcific aortic valve disease; *p < 0.05 versus PM group (one-way ANOVA with Bonferroni post hoc test)

WB assays found that the phosphorylation levels of IKKβ and NK-κB were significantly increased in CAVD valves (Fig. 6a). In order to further investigate the effect of metformin on the NF-κB pathway, we used three different concentrations of metformin for treatment. As expected, metformin significantly reduced the increase of phosphorylated IKKβ and NF-kB induced by PM in a concentration-dependent manner (Fig. 6b). Our further experiments found that 10 μM PDTC could significantly inhibit the protein level of PM-induced osteogenic markers, BMP2 and OPN (Fig. 6c). And ARS staining assays showed that PDTC could reach a good inhibitory effect on calcium deposition (Fig. 6d).

### AKT inhibitor reverses the anti-calcification effect of metformin

**Fig. 7:**
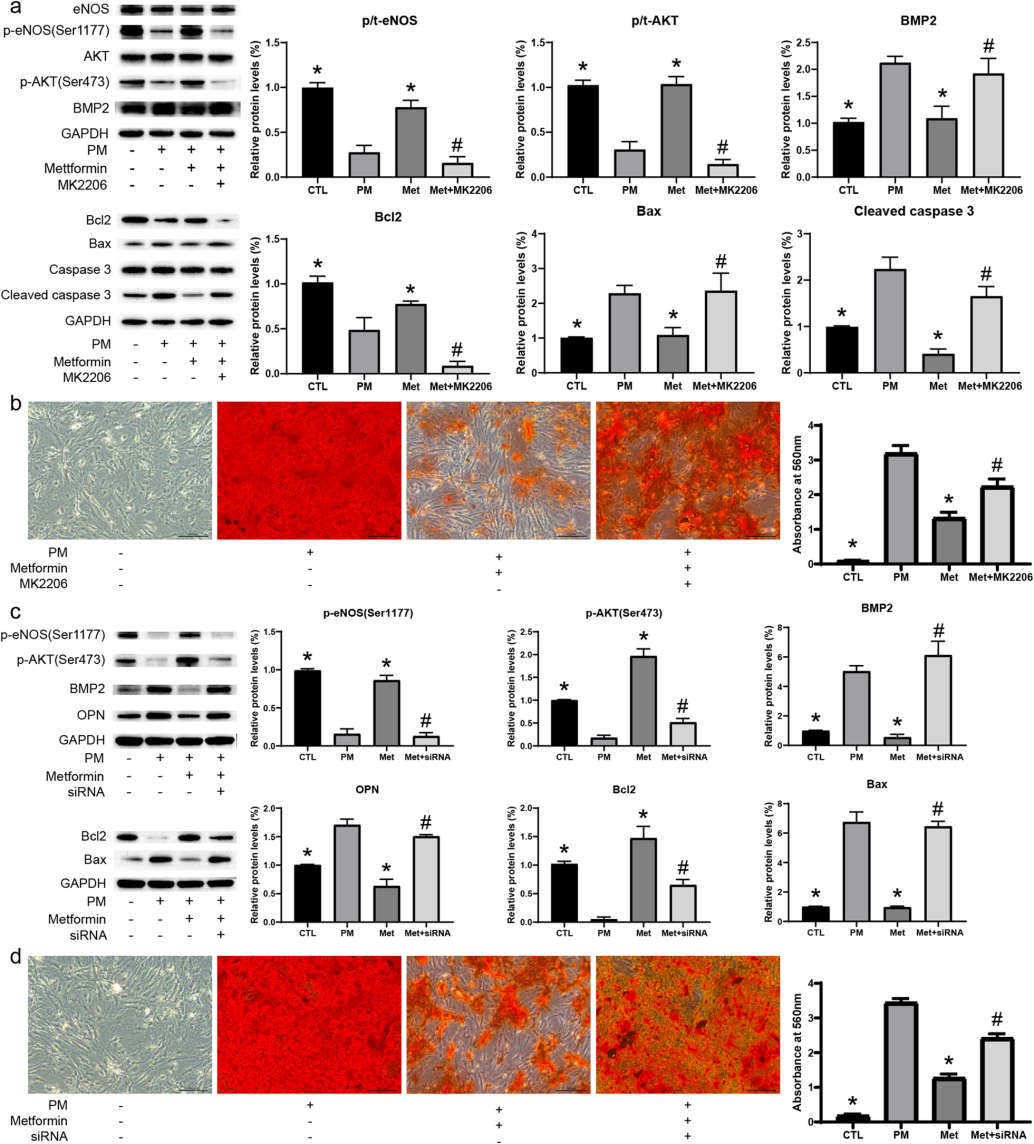
AKT inhibition ameliorates the anti-calcification and anti-apoptosis effect of metformin on AVICs. **a** Protein expression levels of the PI3K/AKT and apoptosis pathway in AVICs after various treatments with metformin (100 μM) and AKT inhibitor (MK2206, 5 μM) for 72 h as determined by WB. **c** Protein expression levels of the PI3K/AKT and apoptosis pathway in AVICs after various treatments with metformin (100 μM) and AKT siRNA for 72 h as determined by WB; **b** and **d** Calcium deposition were detected by ARS Staining after various treatments for 7 days, Scale bar, 200 μm. n = 6 per group. *CTL* control; *PM* phosphate medium; *Met* metformin; *p < 0.05 versus PM group; ^#^p < 0.05 versus Met group (one-way ANOVA with Bonferroni post hoc test)

To further investigate whether the anti-calcification effect of metformin depends on the PI3K/AKT signaling pathway, we used an AKT inhibitor for treatment. According to previous literature, the concentration of MK2206 was determined as 5 μM. MK2206 inhibited the phosphorylated protein level of AKT and eNOS and promoted the expression of osteoblast differentiation marker (BMP2 and OPN) as confirmed by IF (Additional file 1: Fig. S7) and WB assays (Fig. 7a). In addition, ARS staining and calcium deposition quantitative experiments also showed that MK2206 attenuated the anti-calcification effect of metformin (Fig. 7b). Moreover, MK2206 treatment inhibited the anti-apoptotic effect of metformin, reduced the protein level of Bcl2, and increased the expression of Bax and Cleaved caspase 3 (Fig. 7a).

### AKT inhibition by siRNA alleviates the anti-calcification effect of metformin

To further confirm whether the PI3K/AKT signaling pathway plays a key role in AVICs, we used AKT siRNA to inhibit the expression of the PI3K/AKT signaling pathway. We constructed three siRNAs specific to AKT1, and one siRNA with the highest silencing efficiency was screened, confirmed by qRT-PCR and WB (Additional file 1: Fig. S8). AKT siRNA inhibited the anti-apoptotic effect of metformin, reduced the expression level of Bcl2 protein, and increased the protein level of the apoptotic protein Bax, which coincided with decreases in the protein level of phosphorylated AKT and eNOS. Moreover, AKT silencing reversed the inhibitory effect of metformin on osteogenic proteins (BMP2 and OPN) and ameliorated the anti-osteogenic differentiation effect of metformin on AVICs (Fig. 7c). Moreover, AKT siRNA alleviated the anti-calcification effect of metformin, promoted calcium deposition and calcium nodule formation, as confirmed by ARS staining (Fig. 7d).

## Discussion

CAVD is the most common valve disease and the third most common cardiovascular disease; over 2.5 million people are afflicted with aortic stenosis worldwide (Lindman et al. 2016). However, no available medical therapies beyond surgical or transcatheter aortic valve replacement could prevent the relentless progression of CAVD. Regardless of its hypoglycemic effect, metformin may have an effective effect in a variety of cardiovascular diseases, including arrhythmia (Lu et al. 2017), heart failure (Gu et al. 2020), myocardial ischemia–reperfusion injury (Zhang et al. 2020), and atherosclerosis (Jenkins et al. 2018). Metformin can prevent the progression of atherosclerosis and the rupture of atherosclerotic plaques. CAVD has a pathological mechanism similar to atherosclerosis, and Liu et al. indicated an inhibitory effect of metformin on the osteoblastic differentiation of AVICs induced by TGF-β1 by inhibiting β-catenin pathway; however, only few studies have reported the effectiveness of metformin on CAVD (Liu et al. 2018). In this study, we revealed several novel findings. First, we confirmed that metformin could effectively inhibit the calcification of AVICs and the apoptosis and inflammatory response induced by PM. In addition, the PI3K/AKT signaling pathway was involved in the pathological mechanism of CAVD, and inhibition of this pathway induced phenotypic transformation of AVICs.

Metformin can inhibit the differentiation of AVICs into osteoblasts, calcium deposition, and inflammatory factor secretion induced by PM in an AMPK-dependent manner. BMP2 is an effective initiator of osteogenic differentiation of AVICs. OPN is a multifunctional glycoprotein, which is related to various biological processes such as inflammation response and bone remodeling (Small et al. 2017; Yang et al. 2009). Many studies showed the upregulated expression level of BMP2 and OPN in calcific aortic valves, and the expression levels are associated with the degree of aortic calcification (Grau et al. 2012; Mohler et al. 1997; O'Brien et al. 1995). We found that metformin at 100 μM could produce significant inhibitory effect on AVICs calcification and the expression level of BMP2 and OPN. Moreover, inflammation response is closely associated with the occurrence and development of CAVD (Dweck et al. 2013). Inflammatory cells appear before calcium nodules in the aortic valve, and inflammatory cells are subject to localize around calcific nodules (Abdelbaky et al. 2015; Coté et al. 2013). Multiple studies have found that the NF-kB signaling pathway is an important one that regulates the pro-inflammatory response, and it is closely associated with the development of CAVD (Éva Sikura et al. 2021; Liu et al. 2017; Zeng et al. 2017). Our study found that PM induced AVICs to differentiate into osteoblast-like phenotype, thereby secreting more inflammatory factors. However, metformin treatment significantly inhibited the secretion of inflammatory factors, including IL6, IL8, and MCP-1, which coincided with decreases in the protein phosphorylation level of IKKβ and NF-kB. Previous studies also reported that metformin could inhibit the inflammatory response by inhibiting the NF-κB inflammatory signaling pathway (Cameron et al. 2016). These indicate that the anti-calcification effect of metformin may, in part, be due to the inhibition of the inflammatory response by inhibiting the activation of the NF-kB pathway. Moreover, we found that the AMPK inhibitor, compound C, ameliorated the anti-calcification effect of metformin, which suggests that the anti-calcification effect of metformin is dependent on the activation of AMPK.

Metformin significantly inhibited the ROS-mediated oxidative stress of AVICs induced by PM. Multiple studies showed a causal relationship between ROS-mediated oxidative stress and the pathological mechanism of CAVD (Greenberg et al. 2021). ROS, a group of highly active chemical substances produced by mitochondria, is produced by the binding of oxygen and electrons that leaked from the oxidation respiratory chain (Memet et al. 2015). Moreover, ROS, mainly including superoxide and hydrogen peroxide, plays vital roles in cell growth, cell differentiation, apoptosis, and autophagy, etc. (Holmström and Finkel 2014; Zhang et al. 2019) Endothelial NO synthase (eNOS) can produce NO, which is an effective vasodilator and can inhibit AVICs calcification (Miller et al. 2008). In the cardiovascular system, NOS uncoupling and mitochondria are the main sources of ROS production. In the present study, we found significantly increased ROS and decreased phosphorylated eNOS protein in PM-treated AVICs; however, metformin treatment significantly reduced the level of ROS, promoted the protein expression of phosphorylated eNOS, which coincided with decreases of osteogenic transformation markers, including BMP2 and OPN. However, AMPK or AKT inhibition could ameliorate the upregulation effect of metformin on phosphorylated eNOS. Other studies also showed that elevated ROS levels were observed in calcific aortic valves and osteoblast-like AVICs (Branchetti et al. 2013), and the use of NO donor (DETA-NONO) or NO synthesis precursor (L arginine) could effectively inhibit osteogenic differentiation of AVICs (Kennedy et al. 2009; Rattazzi et al. 2020). Taken together, metformin could effectively inhibit PM-induced ROS production dependent on the activation of AMPK and AKT.

The reduced PI3K/AKT signaling pathway is closely associated with the pathological mechanism of CAVD. The PI3K/AKT pathway plays a vital role in cell proliferation, survival, apoptosis, inflammation, and metabolism (Engelman et al. 2006; Hers et al. 2011). Multiple studies showed that PI3K and AKT were decreased in calcific aortic valves, and inhibiting the expression of the PI3K/AKT pathway induced the apoptosis and calcification of AVICs (El Husseini et al. 2014; Parra-Izquierdo et al. 2018). However, this study is the first to determine that metformin could inhibit AVICs calcification by activating the PI3K/AKT signaling pathway. We also found that the expression of the PI3K/AKT pathway was significantly reduced through immunochemical staining and WB assays in calcific aortic valves and PM-induced AVICs, and AKT inhibition abolished the anti-calcification effect of metformin. Moreover, previous studies showed significantly increased apoptosis in calcific aortic valves (Fu et al. 2016). Metformin exerted beneficial effects in multiple cardiovascular diseases, such as heart failure, myocardial reperfusion injury, and atherosclerosis, by mediating apoptosis (Huang et al. 2020; Loi et al. 2019). Consistent with previous studies, our study found that metformin treatment inhibited PM-induced apoptosis of AVICs and promoted the Bcl2/Bax ratio; however, the utilization of AKT inhibitors or siRNA reversed this effect of metformin. These findings indicated that the anti-apoptotic effect of metformin on AVICs was mediated at least in part by activating the PI3K/AKT pathway.

Some limitations in our study must be acknowledged. First, the AVICs were extracted from patients with aortic valve prolapse or severe aortic valve regurgitation, which might differ from normal AVICs. Second, our osteogenic model using PM might not truly simulate the valve calcification process in vivo. Third, the sample size included in this study is relatively small. Finally, we did not determine the effect of metformin in animals, and increased caution should be taken when translating in vitro lab results to in vivo and clinical. Future, more profound studies are warranted to investigate the pathological mechanism of CAVD and the mechanism of metformin in vivo.

## Conclusions

Taken together, this study found that the PI3K/AKT signaling pathway was significantly reduced in calcific aortic valves and PM-induced AVICs. To our knowledge, this study is the first to determine that metformin can inhibit AVICs calcification by activating the PI3K/AKT signaling pathway. Metformin is a promising potential drug in CAVD treatment, and the PI3K/AKT signaling pathway is closely associated with the molecular mechanism of this disease.

## Supplementary Information

**Additional file 1: Figure S1.** Heatmap of DEGs (log2(fold change)). The genes enriched in the PI3K/AKT signaling pathway are indicated by arrows. CTL, control; CAVD, calcific aortic valve disease; DEGs, differentially expressed genes. **Figure S2.** Kyoto Encyclopedia of Genes and Genomes (KEGG) pathway enrichment analysis of differentially expressed genes. **Figure S3.** Gene ontology (GO) functional enrichment analysis of DEGs. GO analysis was conducted using the clusterProfiler package in R software; BP, biological process; CC, cellular component; MF, molecular function; FC, fold change; DEGs, differentially expressed genes. **Figure S4.** Protein-protein interaction (PPI) network of DEGs. PPI network was predicted using the online database, Search Tool for the Retrieval of Interacting Genes (STRING, http://string-db.org/); DEGs, differentially expressed genes. **Figure S5.** AVICs phenotype determination by immunofluorescence staining. Representative immunofluorescence staining images of AVICs showing positive vimentin (green) and α-SMA (red); DAPI (4′,6-diamidino-2-phenylindole) was applied for nuclei counterstaining (blue) (n=6). AVICs, aortic valve interstitial cells; Original magnification, ×40 objective. **Figure S6.** Metformin attenuates AVICs apoptosis by WB assays. The protein expression levels of Bcl2, Bax, and Cleaved caspase 3 in AVICs after phosphate medium (PM) with or without metformin for 72 hours (n=6 per group); AVICs, aortic valve interstitial cells; CTL, control; Met, metformin. **Figure S7.** AKT inhibitor attenuates the anti-calcification effect of metformin. Immunofluorescence staining images of OPN expression in AVICs after phosphate medium (PM) treatment with or without AKT inhibitor (MK2206, 5μM) for 72 hours, DAPI (4′,6-diamidino-2-phenylindole) was applied for nuclei counterstaining (n=4 per group). AVICs, aortic valve interstitial cells; Original magnification, ×40 objective. **Figure S8.** Knockout efficiency of three siRNAs against AKT1. The AKT mRNA and protein expression levels were determined by qRT-PCR and WB (n=6 per group). *, p < 0.05 versus control group. **Table S1.** Characteristics of patients included in the study. **Table S2.** The sequences of three siRNAs targeting AKT1

## Data Availability

All data generated or analysed during this study are included in this published article and its supplementary information files.

## Abbreviations

CAVD: Calcific aortic valve disease
AVICs: Aortic valve interstitial cells
BMP: Bone morphogenetic protein
eNOS: Endothelial nitric oxide synthase
DEGs: Differentially expressed genes
KEGG: Kyoto Encyclopedia of Genes and Genomes
PFA: Paraformaldehyde
IHC: Immunohistochemical
HE: Hematoxylin–eosin
PBS: Phosphate buffer solution
BSA: Bovine serum albumin
TUNEL: Terminal Deoxynucleotidyl Transferase-mediated dUTP Nick-End Labeling
CC: Compound C
IF: Immunofluorescence
CCK8: Cell counting kit-8
OD: Optical density
ROS: Reactive oxygen species
ELISA: Enzyme-linked immunosorbent assay
qRT-PCR: Quantitative real time-PCR
WB: Western blot
ARS: Alizarin Red S
OPN: Osteopontin
PM: Phosphate medium
