# Supplementary material for: Metformin alleviates the calcification of aortic valve interstitial cells through activating the PI3K/AKT pathway in an AMPK dependent way
Source: Mol Med. 2021 Dec 11;27:156. doi: 10.1186/s10020-021-00416-x (PMC8666063; doi:10.1186/s10020-021-00416-x)
Supplement: Supplementary file 1 — Additional file 1: Figure S1. Heatmap of DEGs (log2(fold change)). The genes enriched in the PI3K/AKT signaling pathway are indicated by arrows. CTL, control; CAVD, calcific aortic valve disease; DEGs, differentially expressed genes. Figure S2. Kyoto Encyclopedia of Genes and Genomes (KEGG) pathway enrichment analysis of differentially expressed genes. Figure S3. Gene ontology (GO) functional enrichment analysis of DEGs. GO analysis was conducted using the clusterProfiler package in R software; BP, biological process; CC, cellular component; MF, molecular function; FC, fold change; DEGs, differentially expressed genes. Figure S4. Protein-protein interaction (PPI) network of DEGs. PPI network was predicted using the online database, Search Tool for the Retrieval of Interacting Genes (STRING, http://string-db.org/); DEGs, differentially expressed genes. Figure S5. AVICs phenotype determination by immunofluorescence staining. Representative immunofluorescence staining images of AVICs showing positive vimentin (green) and α-SMA (red); DAPI (4′,6-diamidino-2-phenylindole) was applied for nuclei counterstaining (blue) (n=6). AVICs, aortic valve interstitial cells; Original magnification, ×40 objective. Figure S6. Metformin attenuates AVICs apoptosis by WB assays. The protein expression levels of Bcl2, Bax, and Cleaved caspase 3 in AVICs after phosphate medium (PM) with or without metformin for 72 hours (n=6 per group); AVICs, aortic valve interstitial cells; CTL, control; Met, metformin. Figure S7. AKT inhibitor attenuates the anti-calcification effect of metformin. Immunofluorescence staining images of OPN expression in AVICs after phosphate medium (PM) treatment with or without AKT inhibitor (MK2206, 5μM) for 72 hours, DAPI (4′,6-diamidino-2-phenylindole) was applied for nuclei counterstaining (n=4 per group). AVICs, aortic valve interstitial cells; Original magnification, ×40 objective. Figure S8. Knockout efficiency of three siRNAs against AKT1. The AKT mRNA and prote [file 10020_2021_416_MOESM1_ESM.docx]

Additional Figure S1:


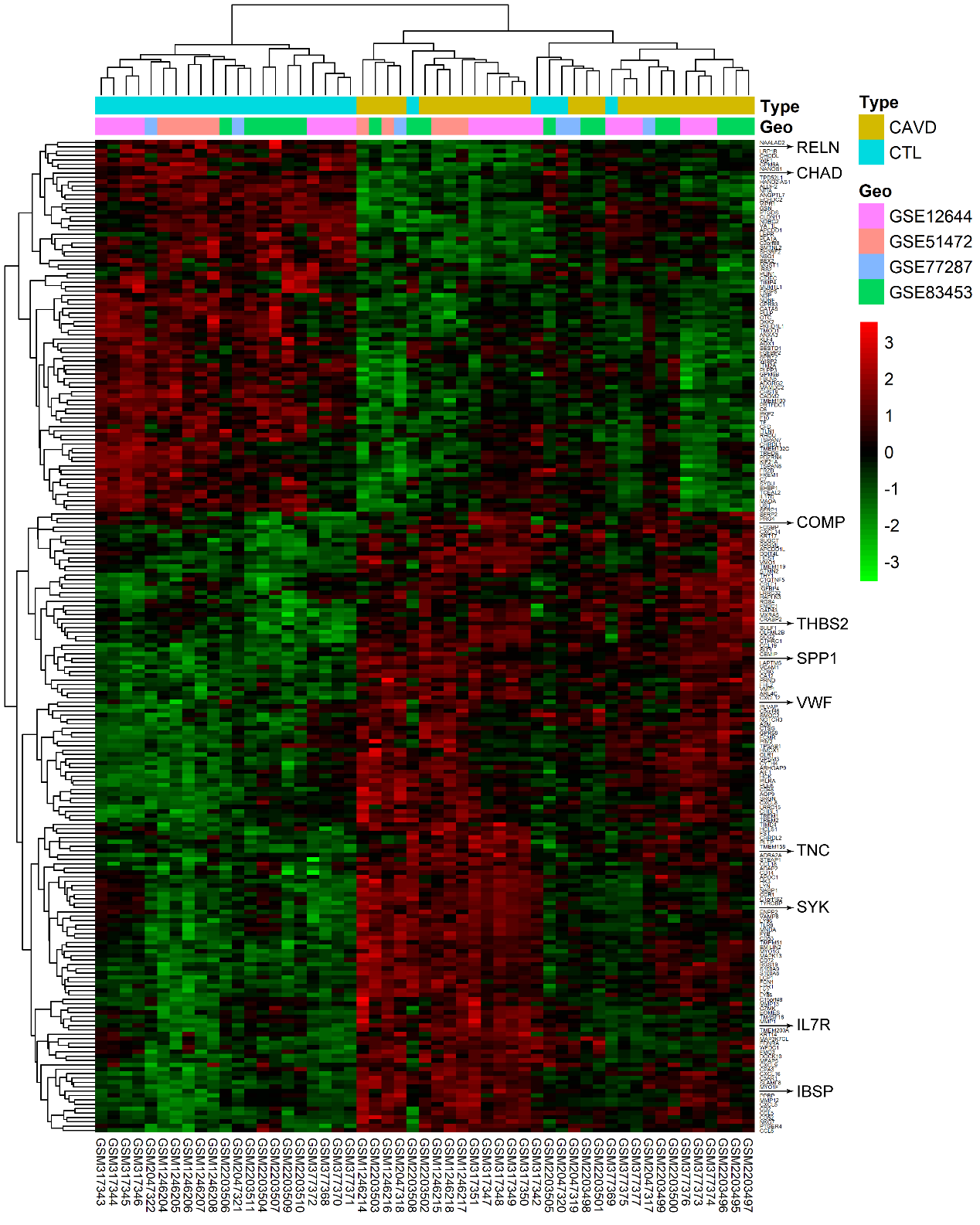


Heatmap of DEGs (log2(fold change)). The genes enriched in the PI3K/AKT signaling pathway are indicated by arrows. CTL, control; CAVD, calcific aortic valve disease; DEGs, differentially expressed genes.

Additional Figure S2:


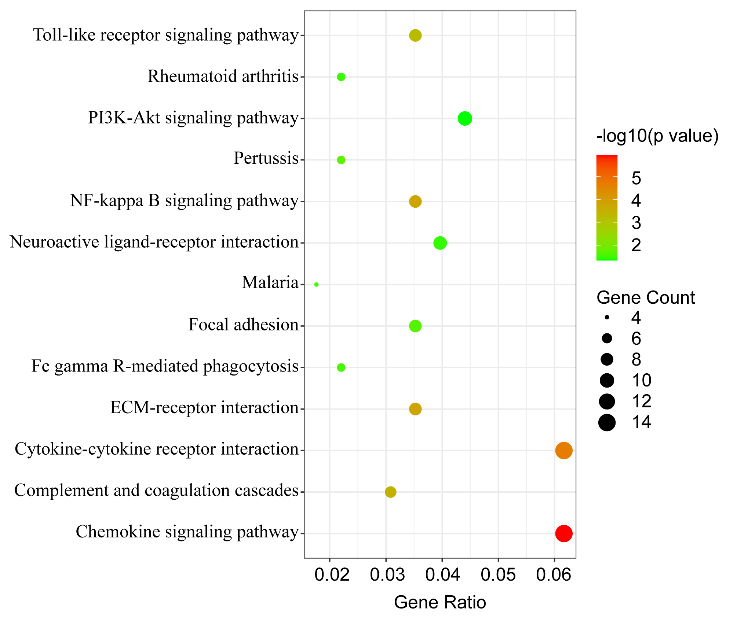


Kyoto Encyclopedia of Genes and Genomes (KEGG) pathway enrichment analysis of differentially expressed genes.

Additional Figure S3:


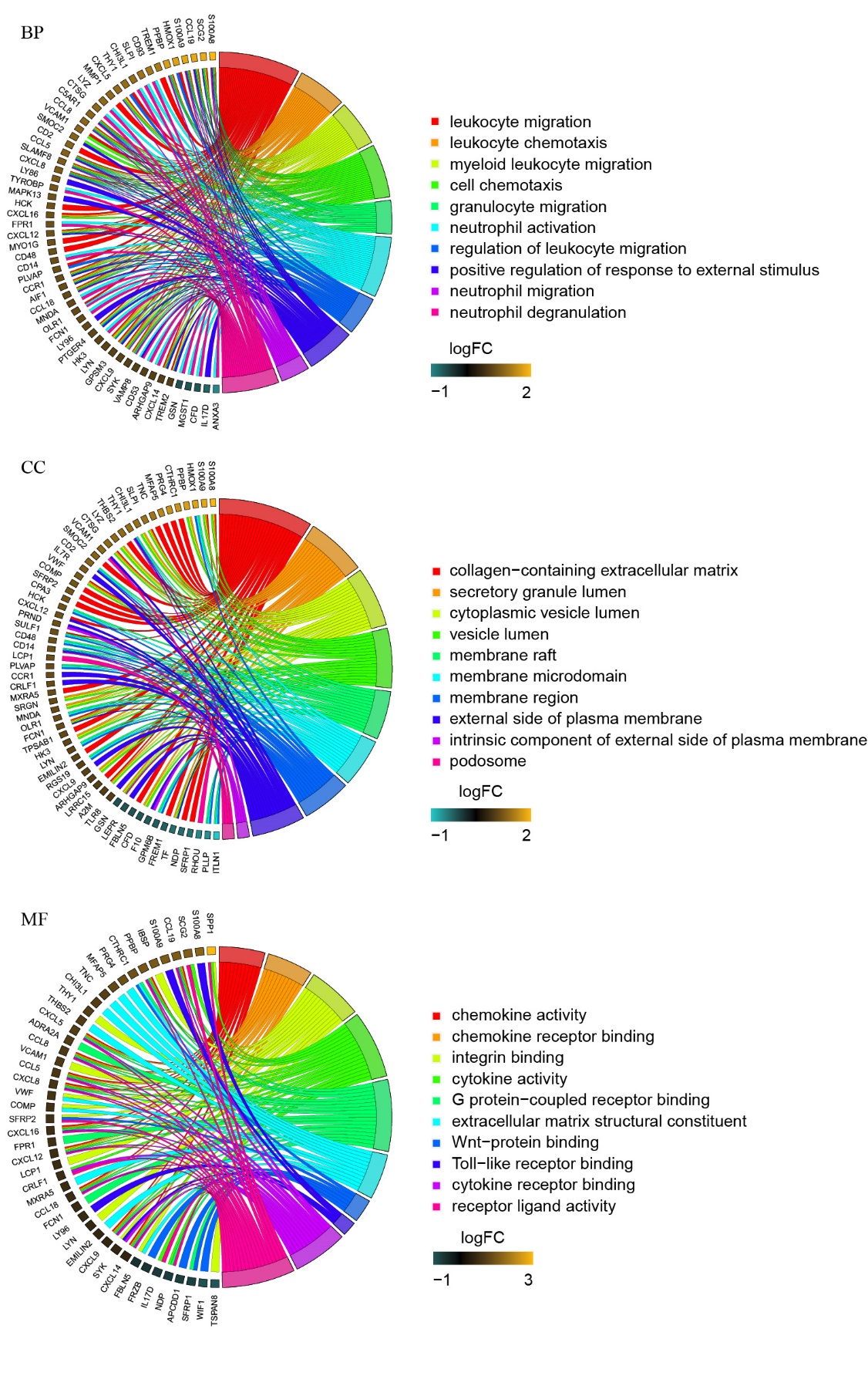


Gene ontology (GO) functional enrichment analysis of DEGs. GO analysis was conducted using the clusterProfiler package in R software; BP, biological process; CC, cellular component; MF, molecular function; FC, fold change; DEGs, differentially expressed genes.

Additional Figure S4:


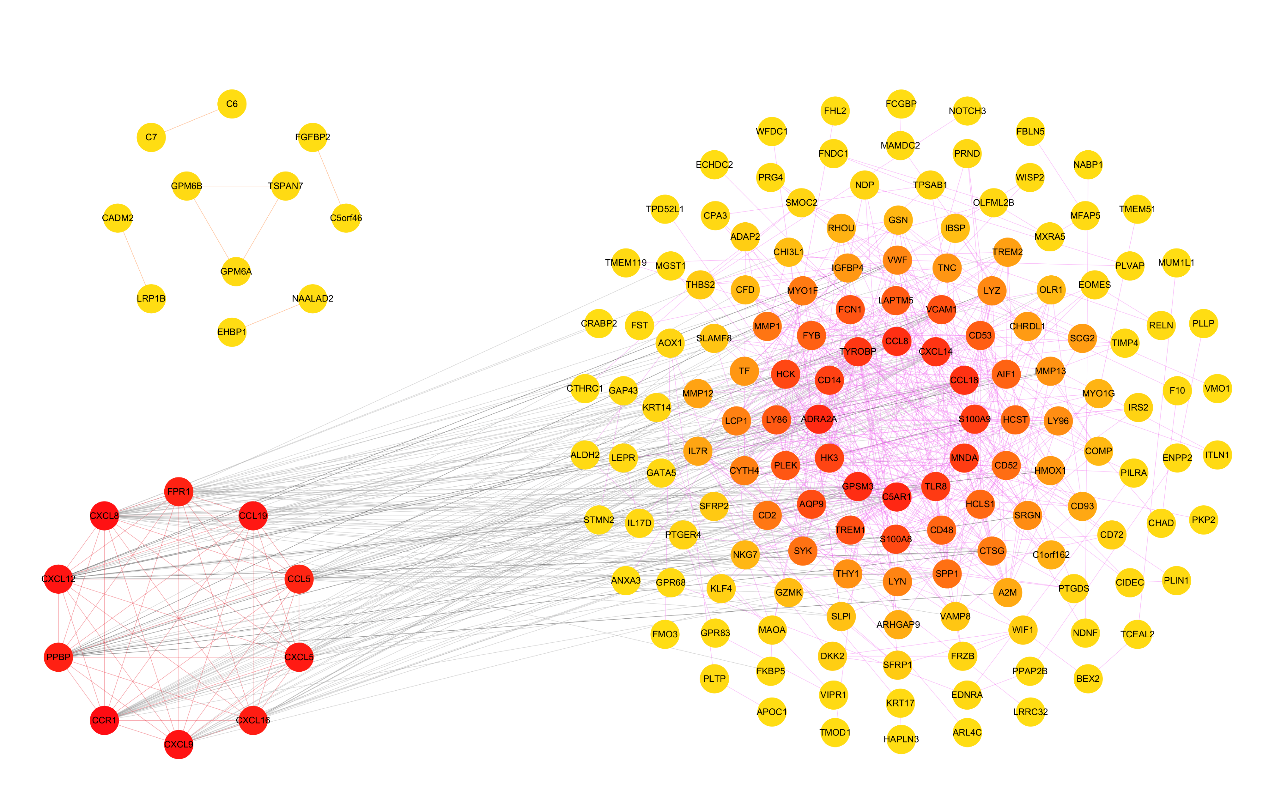


Protein-protein interaction (PPI) network of DEGs. PPI network was predicted using the online database, Search Tool for the Retrieval of Interacting Genes (STRING, http://string-db.org/); DEGs, differentially expressed genes.

Additional Figure S5:


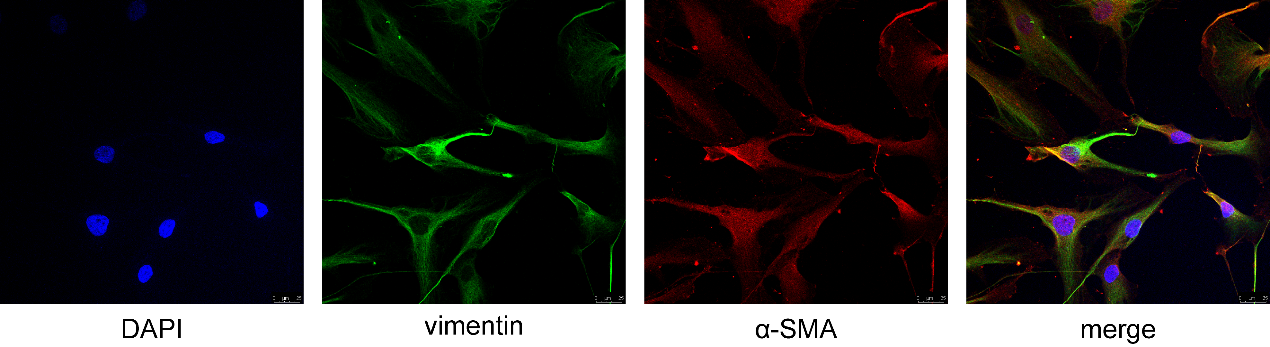


AVICs phenotype determination by immunofluorescence staining. Representative immunofluorescence staining images of AVICs showing positive vimentin (green) and α-SMA (red); DAPI (4′,6-diamidino-2-phenylindole) was applied for nuclei counterstaining (blue) (n=6). AVICs, aortic valve interstitial cells; Original magnification, ×40 objective.

Additional Figure S6:


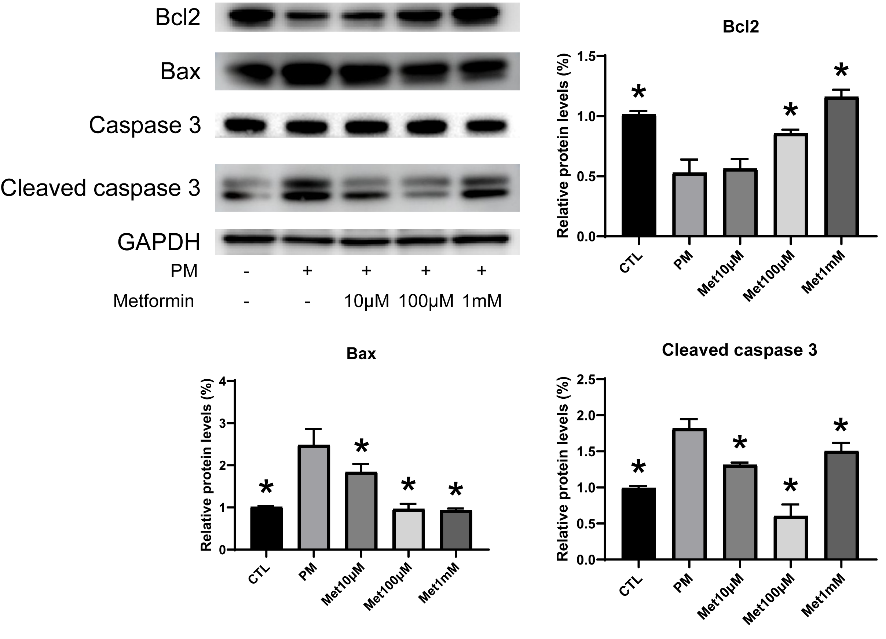


Metformin attenuates AVICs apoptosis by WB assays. The protein expression levels of Bcl2, Bax, and Cleaved caspase 3 in AVICs after phosphate medium (PM) with or without metformin for 72 hours (n=6 per group); AVICs, aortic valve interstitial cells; CTL, control; Met, metformin.

Additional Figure S7:


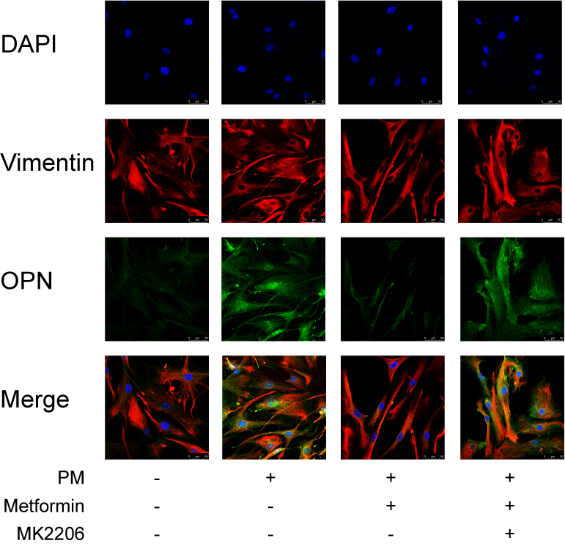


AKT inhibitor attenuates the anti-calcification effect of metformin. Immunofluorescence staining images of OPN expression in AVICs after phosphate medium (PM) treatment with or without AKT inhibitor (MK2206, 5μM) for 72 hours, DAPI (4′,6-diamidino-2-phenylindole) was applied for nuclei counterstaining (n=4 per group). AVICs, aortic valve interstitial cells; Original magnification, ×40 objective.

Additional Figure S8:


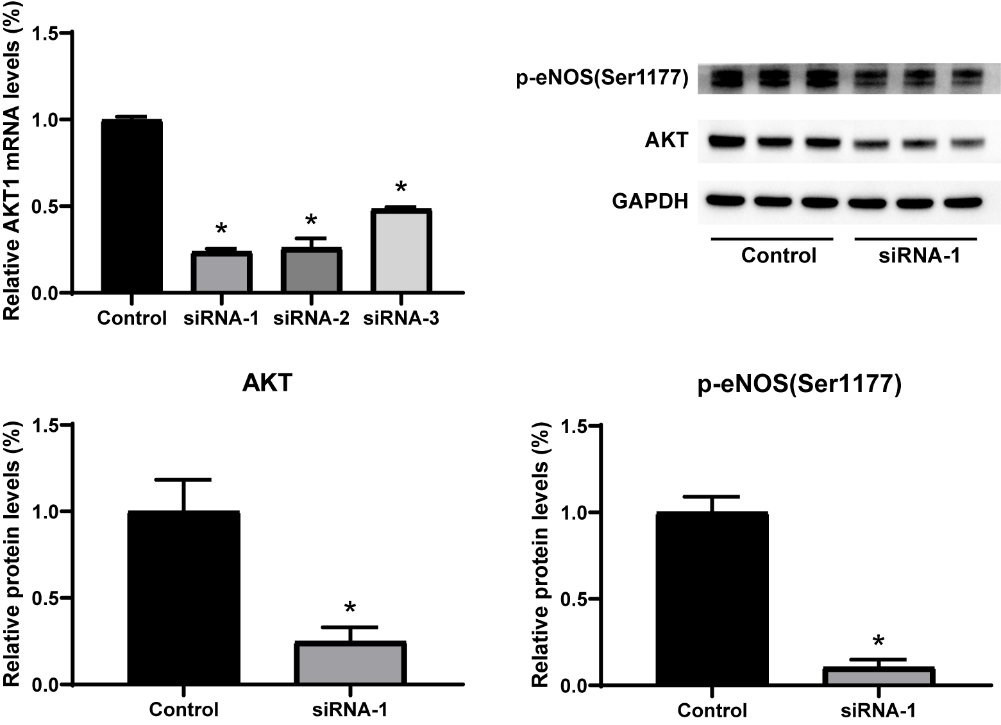


Knockout efficiency of three siRNAs against AKT1. The AKT mRNA and protein expression levels were determined by qRT-PCR and WB (n=6 per group). *, p < 0.05 versus control group

Additional Table S1:

|  | CTL (N=9) | CAVD (N=8) | *p* |
| --- | --- | --- | --- |
| Age, years | 54.1±12.4 | 59.9±7.9 | 0.279 |
| Male/Female | 5/4 | 4/4 | 1.000 |
| Hypertension, % | 0.22 | 0.38 | 0.620 |
| Hyperlipidemia, % | 0.22 | 0.50 | 0.335 |
| Diabetes mellitus, % | 0.00 | 0.25 | 0.206 |
| Diabetic treatment, % | 0.00 | 0.25 | 0.206 |
| LVEF, % | 57.4±11.4 | 63.3±7.0 | 0.233 |
| LVEDD, mm | 62.7±12.3 | 53.5±9.2 | 0.105 |
| BNP, ng/L | 1410.7±2652.7 | 1975.5±2606.8 | 0.665 |
| CKMB, U/L | 10.0±3.4 | 14.1±3.9 | 0.033 |
| ALT, IU/L | 20.2±9.9 | 23.3±16.2 | 0.644 |
| AST, IU/L | 23.1±4.4 | 24.5±5.6 | 0.575 |
| CREA, μmol/L | 93.1±22.8 | 75.4±11.6 | 0.067 |
| BUN, mmol/L | 8.2±3.1 | 5.5±1.5 | 0.037 |

Characteristics of patients included in the study. CTL, control; CAVD, calcific aortic valve disease; LVEF, left ventricular ejection fraction; LVEDD, left ventricular end-diastolic dimension.

Additional Table S2:

| siRNA | sequence |
| --- | --- |
| siRNA-1 | CTCACCCAGTGACAACTCA |
| siRNA-2 | TGAGCGACGTGGCTATTGT |
| siRNA-3 | CCAGGACCATGAGAAGCTT |

The sequences of three siRNAs targeting AKT1.
